# Insights into the Genetic Susceptibility to Type 2 Diabetes from Genome-Wide Association Studies of Obesity-Related Traits

**DOI:** 10.1007/s11892-015-0648-8

**Published:** 2015-09-12

**Authors:** Tugce Karaderi, Alexander W. Drong, Cecilia M. Lindgren

**Affiliations:** Wellcome Trust Centre for Human Genetics, University of Oxford, Roosevelt Drive, OX3 7BN Oxford, UK; Program in Medical and Population Genetics, Broad Institute of Harvard and MIT, Cambridge, MA USA; Big Data Institute, University of Oxford, Oxford, UK

**Keywords:** Obesity, Body mass index, Fat distribution, Waist-to-hip ratio, Adiponectin, Leptin, Non-alcoholic fatty liver disease, Pericardial fat, Subcutaneous fat, Visceral fat, Fat percent, Type 2 diabetes, Genome-wide association study, Insulin resistance, Beta-cell function, Sexual dimorphism

## Abstract

Obesity and type 2 diabetes (T2D) are common and complex metabolic diseases, which are caused by an interchange between environmental and genetic factors. Recently, a number of large-scale genome-wide association studies (GWAS) have improved our knowledge of the genetic architecture and biological mechanisms of these diseases. Currently, more than ~250 genetic loci have been found for monogenic, syndromic, or common forms of T2D and/or obesity-related traits. In this review, we discuss the implications of these GWAS for obesity and T2D, and investigate the overlap of loci for obesity-related traits and T2D, highlighting potential mechanisms that affect T2D susceptibility.

## Introduction

Type 2 diabetes (T2D) is a common metabolic disease of increased plasma glucose levels to which individuals are predisposed to by a combination of genes and environmental factors. The hyperglycemia typically results from decreased insulin sensitivity (insulin resistance) in insulin-dependent tissues (such as skeletal muscle, liver and adipose tissues), which leads to hyperinsulinemia. Subsequently, when the pancreatic beta cells are not capable of producing the amount of insulin required to maintain normal glycemic status, which may be caused by beta-cell dysfunction and/or reduced beta-cell mass, chronic hyperglycemia and T2D occur (reviewed in [1]).

Overall obesity is defined when a person’s body mass index (BMI, weight (in kilograms) divided by height (in meters) squared) is ≥30 kg/m^2^ [2]. Directly measured fat percent (fat%, measured by bioimpedance (BI), dual-energy X-ray absorptiometry (DXA), computerized tomography (CT), or magnetic resonance imaging (MRI)) is a more accurate indicator of adiposity, which also takes the amount of lean and fat mass into account [3]. Other specific measures of individual fat depots and fat distribution include waist circumference (WC), hip circumference (HC), waist-to-hip ratio (WHR), and subcutaneous and visceral adipose tissue (SAT and VAT) [4, 5]. Levels of adiponectin secreted from adipose tissue [6–13], ectopic fat depots such as pericardial fat [14], and non-alcoholic fatty liver disease (NAFLD) [15] are also obesity-related traits.

The prevalence of obesity and T2D is currently escalating worldwide as a consequence of a sedentary lifestyle and increased consumption of high-energy content food [2]. Between 1980 and 2014, the worldwide prevalence of obesity more than doubled. In 2014, 11 % of men (>205 million) and 15 % of women (>297 million) in the world were obese, compared with 5 % for men and 8 % for women in 1980 [16]. The overall prevalence of obesity is at least four times higher in high-income countries compared to that in low-income countries. A similar accompanying increase in the prevalence of T2D is seen as obesity is a risk factor for T2D [17, 18]. In 2014, it was estimated that there are 387 million people living with diabetes (ages 20–79) with a worldwide prevalence of 8.3 %, and ~90 % of these are individuals with T2D. By 2035, this number is expected to increase by 205 million. It is estimated that 77 % of people with diabetes live in low- and middle-income countries [19].

Genetic, but also environmental, factors interact to cause both obesity and T2D as shown by familial aggregation [20–23], family and twin studies on obesity (heritability (*h*^2^)~40–70 %) [22, 24, 25] and T2D (*h*^2^~26–69 %) [21, 26]. Beyond a sedentary lifestyle, socioeconomic status, poor nutrition, infections and differences in the gut flora have also been added to the list of potential environmental triggers of obesity and T2D [27]. Genetic and environmental evidence is also provided by numerous animal studies. Rodent models for T2D, such as the Lep^ob^ and Zucker mice strains rely on the mutations in genes encoding leptin or its receptor to develop T2D via obesity (reviewed in [28]). Evidence of both environmental and genetic effects in an animal model has been shown to exist in the Agouti *A*^*vy*^ mouse, where the obesity phenotype is inherited through an epigenetic effect that is dependent on the maternal diet [29].

Early evidence for the genetic effect in obesity and diabetes was found through linkage studies of monogenic forms of these diseases segregating as Mendelian disorders, in which mutations occurring in a gene lead to extreme and early-onset forms of these conditions. For obesity, these include genes functioning in the leptin-melanocortin pathway, such as the leptin (*LEP*) and melanocortin 4 receptor (*MC4R*) genes (reviewed in [30, 31]). Similarly, monogenic forms of diabetes are caused by mutations in genes such as *GCK*, *HNF4A* and *HNF1A* with allelic series causing maturity onset of the young (MODY) (reviewed in [32]). Linkage studies have been subsequently accompanied by larger and statistically more powerful genome-wide association studies (GWAS) that are designed to dissect the genetic architecture of common complex traits in a hypothesis-free way [33]. GWAS are useful for identifying common genetic variants (i.e. single-nucleotide polymorphisms (SNPs) with a minor allele frequency (MAF) >5 %) that affect a trait outcome or increase the risk of a disease of interest by comparing frequencies of alleles in thousands of individuals, or between cases and healthy controls, respectively. Many variants associated with complex traits and diseases have been discovered so far through the GWAS approach. SNPs that reach genome-wide significance (*p* < 5 × 10^−8^ after correction for multiple-testing, 0.05/1,000,000 independent tests among common variants in the human genome) are specifically targeted for replication and further functional experiments [33]. These associations are important for unraveling biological mechanisms and pathways that might lend themselves to informing about new therapeutic targets.

In this article, we review the current GWAS of obesity-related traits and consider the overlap with T2D-associated loci in order to gain insights into the genetic susceptibility and potential mechanisms that lead to increased risk of T2D.

## Overview of Genetics of T2D and Obesity-Related Traits in the GWAS Era

To date, T2D GWAS efforts including samples of European [34, 35, 36•, 37, 38], East Asian [39–45], South Asian [46, 47], Mexican/Mexican American [48] and African American [49] descent have delivered 76 robust susceptibility loci [50••]. The majority of these T2D associations appear to act through beta-cell function-related pathways. In contrast, a handful of T2D-associated loci seem to primarily operate through insulin resistance (reviewed in [51]). These associated loci generally have small effect sizes and only explain ~6 and ~10–20 % of the variance in disease susceptibility and the heritability, respectively [36•]. Thus, much of the genetic contribution to the disease remains to be discovered.

**Fig. 1:**
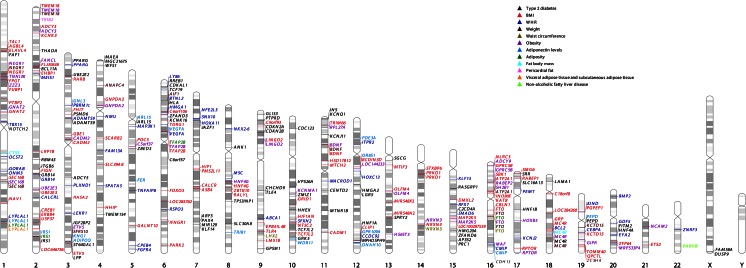
Genome-wide hits for T2D and obesity-related traits loci. GWAS data were obtained from GWAS catalog (http://www.ebi.ac.uk/gwas/, accessed 26 June 2015). Search terms used included obesity, type 2 diabetes, adiposity, waist, fat, body mass index, non-alcoholic liver, adiponectin, weight and adipose. *P* value threshold for association was *p* < 5 × 10^−8^. Associations are labeled with corresponding trait colors

Overall ~185 loci associated with obesity traits have been identified in large-scale GWAS efforts; analyses in Europeans found associations between 77 loci and BMI [52–57, 58••], 48 loci and WHR (adjusted for BMI, WHRadjBMI) [59–61, 62••], and three loci and body fat% [3]. Furthermore, 13 loci were associated with extreme and/or early-onset obesity [63–68], one locus with VAT in women and one locus with VAT/SAT ratio [4], five loci with NAFLD [15], one locus with pericardial fat [14], and seven loci with clinical classes of obesity [69] (Fig. 1). The most recent meta-analysis by the Genetic Investigation of Anthropometric Traits (GIANT) consortium involved 125 studies of European (up to 322,154 individuals) and non-European (up to 17,072 individuals) ancestry [58••, 62••]. Inclusion of non-European ethnicities in these analyses revealed additional genetic associations with 10 BMI loci and a WHRadjBMI locus [58••, 62••]. Ten more associations with BMI were discovered in the secondary analyses of this recent GIANT study [58••]. In other non-European GWAS of BMI and WHRadjBMI, eight additional loci were identified [70–72].

Since the heritability of BMI is 7 % higher at younger ages and increases with the mean age in childhood studies (+1.2 % per year) [73], GWAS of children, adolescents and young adults have been carried out identifying three loci associated with childhood BMI [66, 74–76]. To date, ~2.7 % of the phenotypic variation in BMI was explained by the 97 associations in populations of European and non-European ancestry. Furthermore, common genetic variation (MAF > 5 %) accounted for ~21 % of BMI variation.

## Overlap Between GWAS of Obesity-Related Traits and T2D

### Body Mass Index

The first overall obesity GWAS [54] identified a robust association between BMI and SNPs in the first intron of the fat mass and obesity-associated (*FTO*) gene that has been widely replicated since [70, 72, 77–80]. *FTO* variants had previously been associated with T2D (*p* < 5 × 10^−8^), but this association disappeared after adjusting for BMI, which showed that *FTO* association with T2D is largely due its effect on BMI [54]. In line with this, the effect of *FTO* variants on 23 cardiometabolic traits, in addition to T2D, is mainly mediated through BMI [81]. The *FTO* locus is not only strongly associated with T2D risk [50••] and higher BMI [52] but also increased fasting insulin and homeostatic model estimated insulin resistance (HOMA-IR; *p* = 9.5 × 10^−5^), which is in agreement with insulin resistance playing a part in the *FTO* association with T2D via increased BMI [52]. The FTO protein has been characterized as a 2-oxoglutarate- and Fe(II)-dependent demethylase, possibly involved in mRNA modification, and it is highly expressed in the brain [82, 83]. However, a recent study suggested that the obesity-associated *FTO* variants affect expression of *IRX3*, but not *FTO*, in the human brain, which may mean that *FTO* is not the causal gene in this region. Functional experiments also supported this finding; body weight of *Irx3-*deficient mice was reduced by 25–30 %, and hypothalamic expression of a dominant-negative form of *Irx3* resulted in the same metabolic phenotype as the *Irx3*-deficient mice [84]. The precise biological role of the BMI-associated variants at the *FTO* locus is still unclear and remains to be disentangled.

Following the identification of obesity variants in *FTO*, a robust and replicated association between BMI and variants ~188 kb upstream of *MC4R* was reported [55, 58••, 85–87]. Previous studies have shown that mutations causing MC4R inactivation lead to severe and monogenic forms of obesity [31]. Low frequency variants in *MC4R* were identified in morbid obese individuals (BMI > 40 kg/m^2^) and were associated with obesity [88, 89]. MC4R is a neural G-protein-coupled melanocortin receptor that is highly expressed in the brain [90]. It plays an important role in the regulation of energy balance, specifically in the regulation of energy intake via the control of satiety and energy expenditure (reviewed in [91]).

**Fig. 2:**
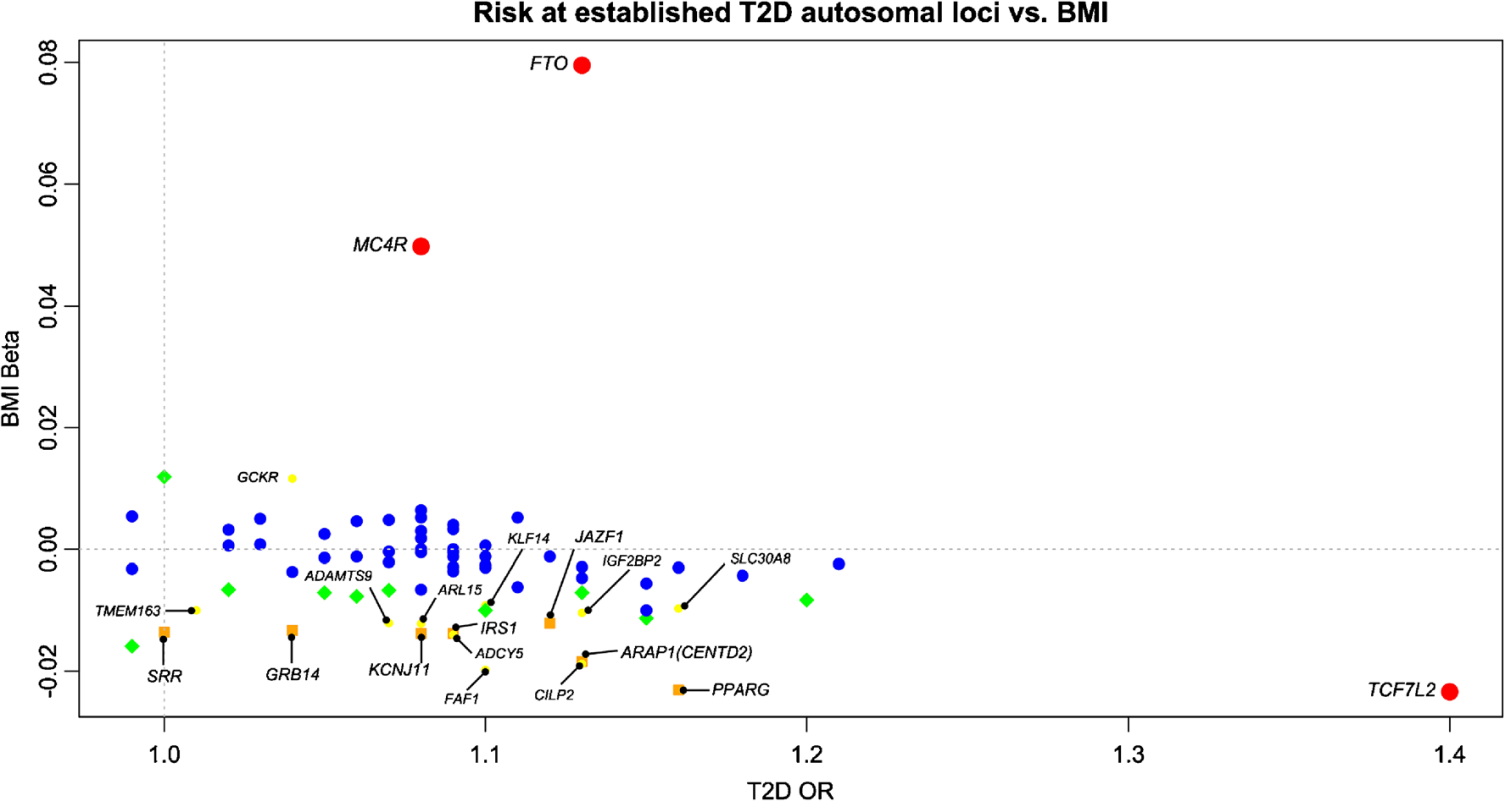
Risk at T2D autosomal loci [50••] vs. BMI [58••]. *P* value thresholds for association with BMI (*y*-axis) are *p* < 5 × 10^−8^ (*red*), 5 × 10^−8^ ≤ *p* < 10^−4^ (*orange*), 10^−4^ ≤ *p* < 0.01 (*yellow*), 0.01 ≤ *p* < 0.05 (*green*) and *p* ≥ 0.05 (*blue*). *Red*, *orange* and *yellow* associations are labeled with corresponding gene names

The *MC4R* locus was associated with both T2D (rs12970134, odds ratio (OR) = 1.08, 95 % confidence interval (CI) = 1.03–1.12, European *p* = 0.0002, trans-ethnic *p* = 2.6 × 10^−8^) [50••] and BMI (beta = 0.05, 95 % CI = 0.043–0.057, *p* = 4.7 × 10^−47^) [58••] (Fig. 2). In addition, another SNP (rs571312) at the same locus, in strong linkage disequilibrium with rs12970134 (*r*^2^ = 0.87, *D*′ = 0.96, HapMap2, Utah Residents with European ancestry population (CEU)), was associated with increased fasting insulin (*p* = 5.2 × 10^−5^), HOMA-IR (*p* = 7.6 × 10^−5^) and T2D (*p* = 0.0004), which is in agreement with insulin resistance playing a part in the *MC4R* association with T2D through BMI [52]. However, in an exome sequencing study of 6760 Pima Indians, mutations decreasing MC4R activity were detected and these individuals with MC4R defects had increased T2D risk, partially independent of BMI in childhood (BMI-adjusted hazard rate ratio = 3.3, 95 % CI = 1.2–9.2, *p* = 0.03). This effect might be due to an increased rate of weight gain compared to adulthood and MC4R affecting downstream insulin signaling [92, 93]. Nevertheless, the effect of *MC4R* variants on T2D risk was completely attributable to BMI in adulthood [94]. Thus, taking into account the changing physiology and hormonal levels during different stages of life would be valuable when considering the biology behind traits and diseases such as BMI and T2D.

The genetic association between T2D and variants in transcription factor 7-like 2 (*TCF7L2*) was first discovered in a candidate gene study [95]. This association was later detected in GWAS, and it is the strongest known association with the disease to date among common variants (rs7903146, OR = 1.4, *p* = 1.9 × 10^−59^) [34, 35, 36•, 37, 38, 50••]. The same lead SNP was also identified in the recent GIANT meta-analysis of BMI (beta = −0.023, *p* = 1.1 × 10^−11^) [58••] (Fig. 2). It is interesting that the T allele of rs7903146 increases T2D risk while decreasing BMI, opposing the idea that increased BMI leads to insulin resistance and T2D. In comparison to *FTO* and *MC4R* variants, *TCF7L2* variants have a much larger effect on T2D risk and a smaller effect on BMI, which might indicate that the *TCF7L2* variants act via T2D to affect BMI (Fig. 2). TCF7L2 is a transcription factor functioning in WNT signaling, which is crucial for cell proliferation, motility, normal embryogenesis, and regulation of myogenesis and adipogenesis (reviewed in [96]). Although the causal variant is still unclear, the T2D risk allele appears to act via lowering the levels of insulin secretion and influencing beta-cell function (reviewed in [51, 96, 97]).

In Fig. 2, 17 genes associated with both T2D [50••] and BMI (orange: *p* < 5 × 10^−8^ ≤ *p* < 10^−4^ for BMI; yellow: 10^-4^ ≤ *p* < 0.01 for BMI) [58••] are shown. These associations provide insights into the genetic overlap of T2D and BMI. For instance, the *ARL15* (rs702634) T2D risk allele was associated with increased fasting insulin (BMI-adjusted, *p* = 5 × 10^–12^), HOMA-IR (*p* = 0.02) and triglyceride levels (*p* = 0.01) as well as decreased high-density lipoprotein (HDL) levels and BMI (*p* = 5.6 × 10^−5^) [50••]. These associations implicate that *ARL15* variants may play a role in insulin resistance leading to T2D susceptibility independently of BMI.

### GWAS of Fat Percent

In a meta-analysis of 15 GWAS with 36,626 individuals of European and Indian Asian descent informative for fat% (as measured by BI and/or DXA), three loci near *FTO*, *SPRY2* and *IRS1* were identified [3]. All of these loci were previously associated with T2D [42, 98]. The fat%-decreasing allele of rs2943650 near *IRS1* was associated with increased risk of T2D as that allele decreased subcutaneous fat but not visceral fat, which is more health damaging (reviewed in [99]). The T2D risk allele of another *IRS1* variant, rs2943640 (*r*^2^ = 0.97, *D*′ = 1.00, HapMap2, CEU), was also nominally associated with decreased BMI (beta = −0.014, *p* = 1.1 × 10^−5^; Fig. 2) [58••]. Furthermore, another variant (rs2943634), strongly correlated with the T2D and fat%-associated rs2943650 (*r*^2^ = 0.80, *D*′ = 0.96, HapMap2, CEU), was associated with fasting insulin levels (beta = 0.025, *p* = 2.5 × 10^−14^) [34, 100, 101]. Insulin receptor substrate 1 encoded by *IRS1* is an important member of the insulin signaling cascade functioning as a docking protein and activating downstream signaling when phosphorylated by the insulin receptor [102]. Given the essential function of IRS1 in insulin signaling and the association of *IRS1* variants with T2D as well as fat% and BMI, this gene is likely to be involved in fat distribution, adipocyte biology and/or insulin resistance [98].

### GWAS of Extreme/Early-Onset Obesity

In the polygenic form of extreme/early-onset obesity, mutations in more than one gene may play a role in susceptibility. Individuals with extreme/early-onset obesity are likely to be enriched for genetic variants predisposing the general population to obesity. Out of five GWAS, only three studies identified novel loci that were not discovered by the previous GWAS of BMI [64, 65, 68]. Except for *FTO* and *MC4R* variants, which affect T2D susceptibility through their effect on BMI, none of these loci overlap with the known T2D loci (Fig. 1) [50••].

### Waist Circumference and Waist-to-Hip Ratio

WHRadjBMI is a measure of fat distribution that indicates the amount of metabolically adverse visceral fat [61], while taking into account the metabolically protective role of gluteal fat [103, 104]. Both WC and WHR are associated with T2D risk independently of BMI [17, 18], and are correlated with the gold standard MRI measurement of central adiposity (i.e. visceral fat, *r*^2^ = 0.6 and *r*^2^ = 0.5, respectively). However, when targeting genetic associations independent of BMI, WHRadjBMI is a better measure of central fat distribution given the strong correlation between WC and BMI (WC-BMI *r*^2^ = 0.9, WHR-BMI *r*^2^ = 0.6) [59].

**Fig. 3:**
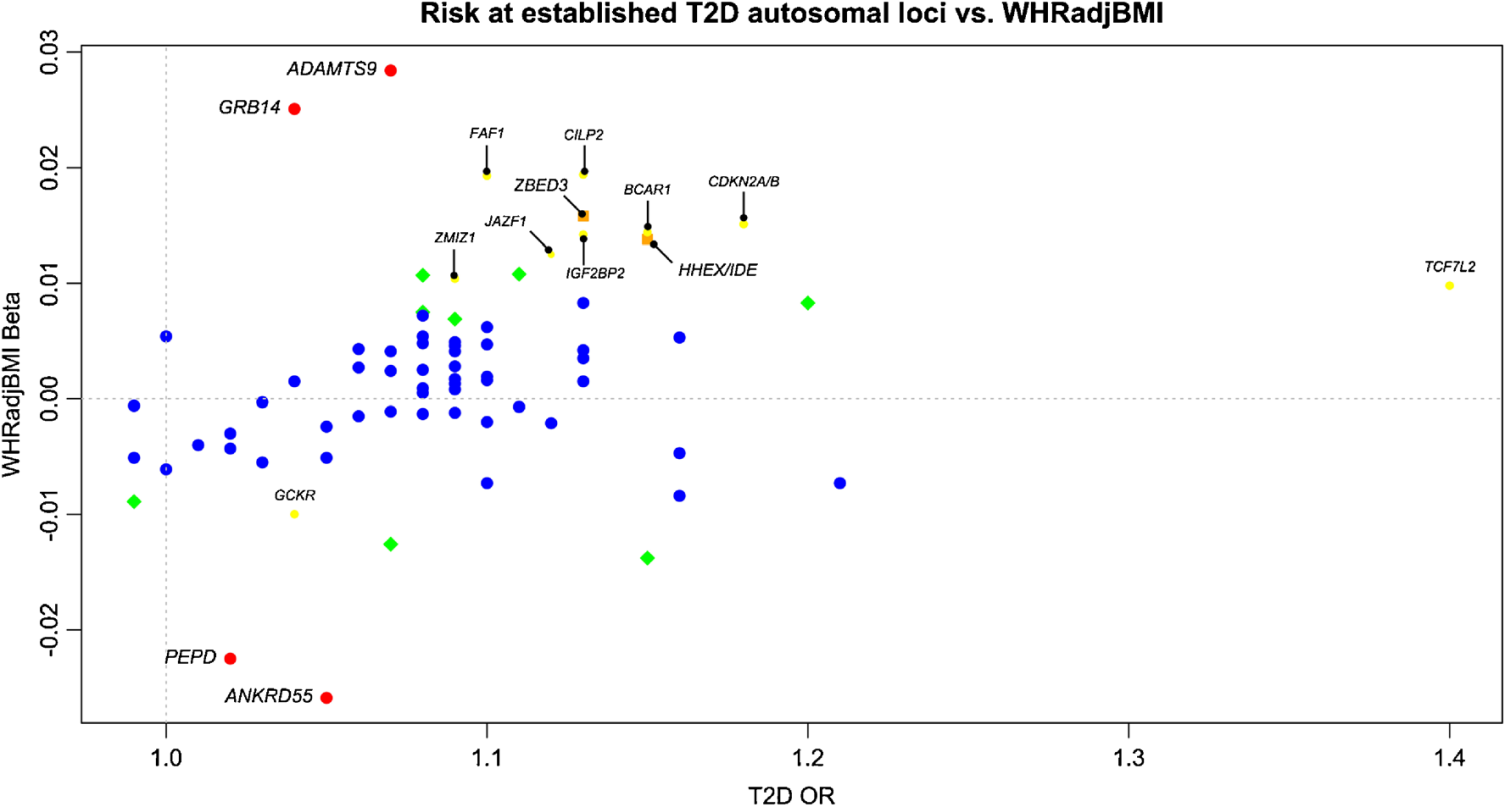
Risk at T2D autosomal loci [50••] vs. WHRadjBMI [62••]. *P* value thresholds for association with WHRadjBMI (*y*-axis) are *p* < 5 × 10^−8^ (*red*), 5 × 10^−8^ ≤ *p* < 10^−4^ (*orange*), 10^−4^ ≤ *p* < 0.01 (*yellow*), 0.01 ≤ *p* < 0.05 (*green*) and *p* ≥ 0.05 (*blue*). *Red*, *orange* and *yellow* associations are labeled with corresponding gene names

A number of variants strongly associated (*p* < 5 × 10^−8^) with T2D risk exhibit opposite directions of effect on BMI and WHRadjBMI (Figs. 2 and 3). For instance, while the T2D risk allele in *GCKR* is associated with increased BMI (rs780094, C allele, beta = 0.01, *p* = 0.0002) [58••] (Fig. 2), the same variant has an opposite effect on WHRadjBMI (rs780094, beta = −0.01, *p* = 0.004) [62••] (Fig. 3). Interestingly, sexual dimorphism was also observed in WHRadjBMI, with a statistically significant (*p* < 0.05) *GCKR* association only in women (rs780094, beta = −0.015, *p* = 0.001). Glucokinase regulatory protein (GCKR) regulates glucokinase (GCK), which is a crucial enzyme for glucose metabolism in the liver and glucose-stimulated insulin secretion from pancreatic beta cells. It was previously observed that over-expression of *GCKR* in the liver significantly improved insulin sensitivity and glucose tolerance in mice resulting in decreased leptin and increased triglyceride levels [105]. This finding may provide a possible explanation for the observed genetic association; the effect of *GCKR* variants may act through leptin to increase BMI, while independently affecting central fat distribution.

Similarly, a pattern of sexual dimorphism was detected for >~40 % (20/49) of the WHRadjBMI lead SNPs, while no pronounced gender difference was found in the BMI and T2D studies [50••, 58••, 62••]. The stronger associations with WHRadjBMI in women were identified in genes that are known to be involved in insulin resistance and/or lipid traits [61, 62••]. One of these genes is *GRB14* (growth factor receptor-bound protein 14; rs3923113, beta = 0.025, *p* = 1.0 × 10^−12^) [62••], which encodes a protein functioning in the regulation of insulin signaling. It binds to insulin receptors, leading to inhibition of their catalytic activity [106]. Female-specific effects of *GRB14* variants were also detected in previous studies of WHRadjBMI and blood lipids [61, 107, 108]. In addition, a gender-specific association with T2D risk was observed with a stronger association in women (rs3923113; OR_men_ = 1.05, *p* = 0.005; OR_women_ = 1.11, *p* = 1.8 × 10^−9^) [36•]. Furthermore, another WHRadjBMI-associated *GRB14* variant (rs10195252, *r*^2^ = 0.79, *D*′ = 1.00, HapMap2, CEU) also exhibited association with decreased BMI (beta = −0.010, *p* = 0.002), HC (beta = −0.021, *p* = 3 × 10^−9^), HDL (*Z*-score = −2.6, *p* = 0.008), increased low-density lipoprotein (LDL) (*Z*-score = 3.5, *p* = 4.5 × 10^−4^), triglycerides (*Z*-score = 5.8, *p* = 7.4 × 10^−9^), fasting insulin (Z-score = 4.6, *p* = 5 × 10^−6^) and HOMA-IR (*Z*-score = 4.8, *p* = 1.9 × 10^−6^). SNP rs10195252 was associated with expression of *GRB14* in SAT as well, indicating that *GRB14* could indeed be the effector transcript in this locus [61].

*GRB14* is an interesting example of a gene with T2D risk alleles causing increased WHRadjBMI and decreased BMI (Figs. 2 and 3). Associations of T2D risk alleles with increased fasting insulin and HOMA-IR implicate *GRB14* variants playing a role in insulin resistance [109]. In rodents and humans, expression of *GRB14* in adipose tissue was negatively correlated with insulin sensitivity. In addition, prolonged fasting and metformin treatment in mice significantly decreased *Grb14* expression in peri-epididymal adipose tissue [110]. Furthermore, improved glucose homeostasis and enhanced insulin signaling were observed in *Grb14*-deficient mice [111]. These findings provide evidence for the importance of *GRB14* regulation in insulin resistance and show that complete understanding of its regulation is essential for identification of new therapeutic pathways in obesity and T2D [112].

ADAMTS9 is a member of the ADAMTS (a disintegrin and metalloproteinase with thrombospondin motifs) family of proteins involved in cleaving proteoglycans, controlling organ maturation and development as well as inhibiting angiogenesis. Its expression is high in all fetal tissues, adult heart and skeletal muscle [113]. Similar to *GRB14*, *ADAMTS9* is also implicated in insulin sensing [61], and variants within this gene show a stronger WHRadjBMI association in women (rs6795735, beta = 0.025, *p* = 9.8 × 10^−14^ [61]; rs2371767, *p*_men_  = 0.008, *p*_women_  = 7.1 × 10^−23^ [108]; rs6795735-rs2371767 *r*^2^ = 0.31, *D*′ = 1.00, HapMap2, CEU). Furthermore, the WHRadjBMI-associated variant (rs6795735) was also nominally associated with decreased HDL (rs6795735, *Z*-score = −2.5, *p* = 0.01) and T2D risk (OR = 1.12, *p* = 0.002), but not with BMI (Fig. 3) [50••, 61]. ADAMTS9 seems to play a role in insulin resistance in peripheral tissues [114]. Although a possible association of T2D risk allele with beta-cell function has also been reported [115], it was not detected in larger GWAS [36•].

*ANKRD55-MAP3K1* is another T2D susceptibility locus (rs459193, OR = 1.05, *p* = 0.03 ) [50••] that was also associated with WHRadjBMI (beta = −0.026, *p* = 1.6 × 10^−11^) [62••] but not overall obesity (Fig. 3). Due to lack of association of the lead SNP (rs459193) with *ANKRD55* expression, *ANKRD55* may not be the functional gene in this region. The neighboring gene *MAP3K1*, with known functions in insulin signaling, has been suggested as a mediator of the biological effect [36•, 101], but that remains to be validated.

*PEPD* encodes peptidase D, which is an enzyme functioning in the recycling of proline and potentially in collagen production. Variants near *PEPD* were associated with fasting insulin (rs731839, BMI-adjusted beta = 0.015, *p* = 5.1 × 10^−12^) [100, 101] and adiponectin levels (rs731839, beta = −0.03, *p* = 8 × 10^−12^) [7], which is directionally consistent with the function of adiponectin in regulating insulin sensitivity. Furthermore, the intronic variant rs3786897 (rs3786897-rs731839 *r*^2^ = 0.34, *D*′ = 1.00, HapMap2, CEU) was nominally associated with T2D susceptibility (European OR = 1.02, *p* = 0.3, 12,171 cases and 56,862 controls; East Asian OR = 1.17, *p* = 3.5 × 10^−7^, 6952 cases and 11,865 controls; trans-ethnic *p* = 3.3 × 10^−4^ ) [50••] and WHRadjBMI (beta = −0.022, *p* = 9.7 × 10^−11^; Fig. 3) [62••]. Interestingly, SNP rs8182584 is strongly correlated with the fasting insulin and adiponectin-associated variant at this locus (rs731839-rs8182584 *r*^2^ = 0.82, *D*′ = 0.92, HapMap2, CEU) and was also associated with reduced expression levels of *PEPD* in adipose tissue (beta = −0.13, *p* = 9.96 × 10^−10^) [7]. The effect of *PEPD* variants may be mediated through insulin and/or adiponectin pathways. Even though *PEPD* associations with T2D are ethnically heterogeneous, identification of a possible role of *PEPD* in susceptibility to T2D and obesity may provide crucial insights into biological mechanisms of these conditions.

### GWAS of Other Obesity-Related Traits: Abdominal Subcutaneous and Visceral Adipose Tissue, Non-alcoholic Fatty Liver Disease and Pericardial Fat

Similar to WHRadjBMI associations, sexual dimorphism was observed in genetic associations with SAT and VAT, highlighting the importance of physiological and hormonal difference in susceptibility to obesity and T2D in men and women [4]. In a GWAS of NAFLD, associations with five loci were identified [15]. One of these loci, the previously reported WHRadjBMI locus at *LYPLAL1*, was also associated with VAT/SAT ratio in women (rs4846567, *p* = 0.0004) [4] and T2D (rs2820446, *p* = 2.3 × 10^−6^; rs4846567-rs2820446 *r*^2^ = 1.00, *D*′ = 1.00, HapMap2, CEU) [50••]. Women are known to have more subcutaneous fat but less visceral fat compared to men [5]. Given the protective role of subcutaneous fat in T2D susceptibility, it is plausible to observe more men with T2D. Globally, the prevalence of T2D is higher in men, but the reasons for this observation may not be limited to the amount of subcutaneous fat in men [116]. In a GWAS of pericardial fat, only one locus (*TRIB2*) reached genome-wide significance, but this locus is also devoid from associations with T2D and other obesity traits (Fig. 1). This lack of overlap between loci associated with T2D, the more commonly used obesity trait measures (BMI, WHRadjBMI, etc.) and the other obesity-related traits such as pericardial fat may indicate that there is a different genetic architecture for pericardial fat and potentially for other ectopic fat depots. Anthropometric and more specifically measured traits might be more distinct than the close relationships between these phenotypes indicate, or these observations most likely reflect that there is a power difference in detection of loci between these studies [14].

### GWAS of Adiponectin Levels

Adiponectin is an adipokine secreted by adipocytes increasing insulin sensitivity [117–119]. Adiponectin levels are positively correlated with HC and inversely correlated with WC, WHR, body fat%, BMI, T2D and coronary heart disease [120–124]. Levels of adiponectin are highly heritable (30–70 %) [125–127], and a number of GWAS have been performed to identify genes affecting adiponectin levels [6–13]. The previously discussed T2D/BMI-associated *ARL15* locus also showed an independent association with adiponectin levels (rs702634-rs4311394 *r*^2^ = 0.09, *D*′ = 0.90, HapMap2, CEU). The lead SNP at the *ARL15* locus (rs4311394) was associated with lower adiponectin levels (*p* = 2.9 × 10^−8^), increased T2D risk (OR = 1.11, *p* = 3.2 × 10^−3^) and coronary heart disease (OR = 1.12, *p* = 8.5 × 10^−6^) [13]. The function of ARL15 is not known, but *ARL15* expression is more pronounced in skeletal muscle [13]. Interestingly, glucose is disposed in skeletal muscle in an insulin-dependent manner, and adiponectin trafficking is essential for insulin sensitivity and glucose transport in muscle. ARL15 is structurally similar to proteins functioning in intracellular vesicle trafficking, and it was suggested that it might play a role in insulin signaling and glucose transport [128, 129]. Therefore, effects of *ARL15* variants may be mediated via insulin resistance and/or adiponectin trafficking [13].

## Conclusions

T2D loci appear to affect susceptibility to T2D via two main mechanisms: (1) through insulin resistance, i.e. insulin sensitivity (measured by fasting insulin and HOMA-IR) and/or (2) through a beta-cell dysfunction (measured by fasting glucose and homeostatic model estimated beta-cell function). In addition, these loci, in general, also exhibit overlapping associations with obesity-related traits and blood lipid levels (HDL, LDL, triglycerides), which might explain the phenotypic overlap with obesity and cardiovascular diseases. However, these associations are often heterogeneous and variants may have opposite directions of effect for different obesity-related traits, reflecting the intricate biology behind them. For instance, most T2D risk alleles seem to be associated with a decrease in BMI, except for the variants in *FTO*, *MC4R* and *GCKR*, two of which are known to affect T2D susceptibility through BMI [50••]. In contrast, most T2D risk variants are associated with increased WHRadjBMI (Figs. 2 and 3). This observation might indicate distinct mechanisms by which (1) WHRadjBMI- and BMI-increasing alleles act on T2D risk, and (2) T2D risk alleles act on BMI. Increased BMI and central adiposity (defined by increased WHRadjBMI) are known to be health damaging and raising T2D risk via insulin resistance. However, there seems to be a second mechanism where risk alleles (e.g. *TCF7L2* variants) predominantly act via T2D and decrease BMI, not vice versa. More targeted genetic and functional studies are necessary to explore these mechanisms and biological pathways implicated (reviewed in [130, 131]).

The heritability of obesity and T2D is not entirely explained by all the loci discovered so far [36•, 50••, 52, 58••, 61, 62••]. More studies with larger sample sizes, in different ethnicities, employing various approaches such as rare variant analysis, exome sequencing, studies of epigenetics and gene-environment interactions are necessary to help explain the missing heritability. Identifying actual functional variants may also increase the phenotypic variance explained by the known loci. Identification of novel loci and functional variants is also required to gain a better understanding of the genetic architecture of body shape, fat depots and T2D. Discovery of additional overlapping genetic associations could provide important insights into the role played by obesity in susceptibility to T2D.

Beyond filling out the gaps in the heritability estimates, deciphering biological mechanisms and pathways that mediate effects leading to susceptibility to obesity and T2D is essential for development of new therapeutic strategies, including lifestyle changes. It is noticeable that genes within loci that are BMI- and WHRadjBMI-associated display different gene expression patterns; they have higher expression levels in the hypothalamus and adipose/peripheral tissues, respectively [52, 61]. These initial observations were further supported by the evidence from Data-driven Expression Prioritized Integration for Complex Traits (DEPICT) analyses in the recent GIANT BMI and WHRadjBMI meta-analyses [58••, 62••]. For WHRadjBMI, significant pathways and gene sets included adiponectin signaling, insulin sensitivity and regulation of glucose levels, skeletal growth, transcriptional regulation and those functioning in the development of metabolically active tissues such as adipose, liver and muscle [62••]. In contrast, highlighted pathways and gene sets for BMI included those functioning in the central nervous system involved in synaptic function, long-term potentiation and neurotransmitter signaling [58••].

Monogenic obesity genes in the leptin-melanocortin pathway provide the link between adipose tissue and the hypothalamus, which are critical sites for balancing the energy need of the body. Genes functioning in the leptin-melanocortin pathway such as those encoding leptin (*LEP*), leptin receptor (*LEPR*), melanocortin 4 receptor (*MC4R*), pro-opiomelanocortin (*POMC*) and brain-derived neurotrophic factor (*BDNF*) have been implicated in the monogenic form of obesity (reviewed in [30]). Leptin is a hormone produced by adipocytes that play an important role in food intake and weight regulation. Increased leptin signaling in the hypothalamus leads to decreased food intake via MC4R and POMC-derived peptide alpha-melanocyte stimulating hormone (alpha-MSH). Many of the monogenic obesity genes lie within loci that are also associated with T2D [50••]. The overlap between monogenic obesity genes and obesity genes identified via GWAS (e.g. *MC4R* and *BDNF*) might imply a role of hypothalamic dysfunction affecting the regulation of energy balance in polygenic obesity, which can drive T2D.

Gender-specific effects are observed for anthropometric traits, particularly for waist-related phenotypes, and understanding their biological influences is crucial [61, 108]. For instance, variants in and around *PPARG* have been associated with T2D, monogenic obesity and WHRadjBMI. Of these, the WHRadjBMI association exhibited sexual dimorphism with a significantly stronger effect in women (beta_women_ = 0.035, beta_men_ = 0.005) [62••]. In parallel with that, gender differences were detected in response to PPARG-agonist therapy in patients with T2D which might indicate different mechanisms for insulin resistance in men and women [132]. Even though biological functions of associated loci are not clear for many genes, gender-specific effects are detected during/after puberty and are potentially attributable to sex hormones [133]. In addition, distribution of body fat also affects metabolic pathways, and body fat has an endocrinological role producing hormones such as estrogen, progesterone, leptin and adiponectin, which affect the regulation of energy balance in the hypothalamus and insulin sensitivity [134]. The understanding of sexual dimorphisms is likely to improve exploration of metabolic disease processes and design of better therapeutic approaches.

In summary, the recent GWAS of obesity-related traits and T2D show considerable overlap in associated loci. These identified associations point to potential mechanisms through which obesity traits affect T2D susceptibility.
